# Draft Genome Sequences of the Turfgrass Pathogen *Sclerotinia homoeocarpa*

**DOI:** 10.1128/genomeA.01715-15

**Published:** 2016-02-11

**Authors:** Robert Green, Hyunkyu Sang, Taehyun Chang, Elisha Allan-Perkins, Elsa Petit, Geunhwa Jung

**Affiliations:** a Stockbridge School of Agriculture, University of Massachusetts, Amherst, Massachusetts, USA; b School of Ecology & Environmental System, Kyungpook National University, Sangju, Gyeongbuk, South Korea

## Abstract

*Sclerotinia homoeocarpa* (F. T. Bennett) is one of the most economically important pathogens on high-amenity cool-season turfgrasses, where it causes dollar spot. To understand the genetic mechanisms of fungicide resistance, which has become highly prevalent, the whole genomes of two isolates with varied resistance levels to fungicides were sequenced.

## GENOME ANNOUNCEMENT

*Sclerotinia homoeocarpa*, the causal agent of dollar spot disease, is the most economically important cool-season turfgrass pathogen. This sterile ascomycete fungus has developed cross-resistance and multiple resistance to the demethylation inhibitor (DMI), methyl benzimidazole carbamates (MBC), and dicarboximide fungicide classes (1–4). Two isolates of *S. homoeocarpa*, HRS10 and HRI11, were collected from individual symptomatic leaf blades of creeping bentgrass (*Agrostis stolonifera*) in 2009 (5). HRI11 displays resistance to DMI fungicides and other chemically unrelated fungicide classes, while HRS10 is sensitive to all fungicide classes (6, 7).

The isolates were preserved on oat seeds (*Avena sativa* L.), according to methods described previously (8). Both isolates were transferred to potato dextrose agar (Difco Laboratories, Detroit, MI), subcultured after 4 days onto potato dextrose broth, and grown for an additional 4 days. Mycelia were freeze-dried using a lyophilizer (Labconco, Kansas City, MO), and the total genomic DNA was extracted using a modified cetyltrimethylammonium bromide method (9).

Libraries were made using a combination of PacBio CLR reads with 20-kb inserts at the High-Throughput Sequencing Facility (HTSF) at North Carolina University, USA, and 2 × 100 Illumina HiSeq reads (Macrogen, Seoul, South Korea). Over 38,000,000 Illumina HiSeq and 1,420,000 PacBio CLR reads were generated for each isolate. The Illumina reads were polished using default parameters with Trimmomatic (10). *De novo* assembly was performed using SPAdes version 3.6.1 (11), with k-mer lengths of 33, 55, and 77. Contigs >5,000 bp and PacBio CLR reads were used for scaffolding with SSPACE-LongRead (12). Scaffolding upgraded the HRS10 draft genome to 231 scaffolds and a total length of 42,266,283 bp (*N*_50_, 600,417 bp), with a largest scaffold of 1,672,908 bp and a G+C content of 43.35%. The HRI11 assembly was upgraded to 257 scaffolds and a total length of 43,359,131 bp (*N*_50_, 709,078 bp), with a largest scaffold of 1,993,158 bp and a G+C content of 43.83%.

Repeats were masked using RepeatMasker and the fungal repetitive elements database from Repbase (13). A total of 1,272,222 bp were masked in HRS10 (3.01%), and 1,353,753 bp were masked in HRI11 (3.12%). Using the masked genomes, 12,216 and 12,912 proteins were annotated for HRS10 and HRI11, respectively, using RNA transcripts from *S. homoeocarpa* (6) as cDNA hits with Augustus (14).

Previous reports (6, 7) have revealed multiple mechanisms of fungicide resistance conferred in isolates HRS10 and HRI11. ATP-binding cassette (ABC) transporters have been shown to be upregulated in the absence and presence of fungicides and contribute to insensitivity to multiple fungicidal classes. Moreover, zinc finger proteins and other transcription factors play a potential role in the detoxification of xenobiotic substances in *S. homoeocarpa* (6). These transcription factors and their DNA-binding domains can now be searched on a genome-wide level for comparisons between HRS10 and HRI11.

### Nucleotide sequence accession numbers.

This whole-genome shotgun project has been deposited in GenBank under the accession no. LNGN00000000 for HRS10 and no. LNKV00000000 for HRI11. The versions described in this paper are the first versions, LNGN01000000 and LNKV01000000, respectively.
